# Rethinking residual p53 function in HPV-positive cervical cancer cells

**DOI:** 10.1128/jvi.00479-25

**Published:** 2026-05-27

**Authors:** Sannia Farrukh, Nezka Kavcic, Giulia Canarutto, Silvano Piazza, Lawrence Banks

**Affiliations:** 1 International Centre for Genetic Engineering and Biotechnology 18470, Trieste, Italy; College of Agriculture & Life Sciences, University of Arizona, Tucson, Arizona, USA

**Keywords:** p53, gain-of-function, HPV, cervical cancer

## Abstract

Human papillomavirus (HPV)-driven cancers remain some of the most prevalent cancers globally. HPV E7 induces unscheduled cellular DNA replication in the infected cell; the resulting apoptotic signaling is counteracted by high-risk HPV E6 oncoprotein, which promotes cell proliferation by targeting many cellular proteins for ubiquitin-mediated proteasomal degradation, including the p53 apoptotic tumor suppressor. E6 oncoprotein forms a heterodimeric complex with the ubiquitin ligase E6AP to recruit and degrade p53, inhibiting pro-apoptotic cellular signaling and disrupting cell cycle progression, leading to increased tumor cell growth. In addition, E6 oncoproteins bind p53, inhibiting its transcriptional transactivation function and disrupting the activation of the p21-Waf1-Cip1 pathway. However, residual p53 activity has been reported in HPV-positive cell lines, indicating a potential gain-of-function role for p53 in HPV-positive cervical cancer cells. To examine this, we used CRISPR-Cas9 ribonucleic protein complex to generate an HPV-positive p53 knockout (KO) HeLa cell line. We observed that the growth and metabolic activity of p53 KO cells are severely reduced. Transcriptomic analysis of these cells revealed gain-of-function (GOF) p53 activities and enrichment of wild-type p53 pathways. Immunoprecipitation analysis confirmed a pool of GOF p53 in these transformed cells. This study thus re-emphasizes the question of the role of residual p53 and provides a model system to investigate its functional mechanism in HPV-positive cervical cancer cells.

**IMPORTANCE:**

Cervical cancer is the fourth most common cause of cancer in women globally, with an estimated 660,000 new cases yearly, and is one of the leading causes of cancer death, with around 350,000 deaths worldwide in 2022. One of the key degradation targets of human papillomavirus (HPV) is the p53 tumor suppressor; however, residual amounts of the protein are still detected in HPV-positive cells. In this study, we created a p53-knockout cell line to determine if residual p53 has any gain-of-function growth-promoting activities. Our results demonstrate that cells without p53 grow slower, indicating that residual p53 has tumor-promoting functions and a conformation like that of mutant p53 when in complex with E6, indicating alternative mechanisms by which HPV can subvert the function of p53.

## INTRODUCTION

Over 200 human papillomavirus types have been described; they are epitheliotropic viruses that infect either cutaneous or mucosal epithelia, mostly causing benign and imperceptible infections (1). High-risk human papillomavirus (HPV) types, mainly HPV16 and HPV18, are associated with the development of human cancers (2). Upon infection, the virus expresses the E7 oncoprotein to promote the S phase, increasing the pool of cells undergoing DNA replication (3). Normally, these differentiating epithelial cells would undergo growth arrest or apoptosis in response to this unscheduled DNA replication, but the high-risk HPV E6 oncoprotein promotes cell proliferation by targeting multiple host cell proteins, including the p53 tumor suppressor, for ubiquitin-mediated proteasomal degradation (4). Persistent expression of these viral oncoproteins leads to the development of cancer and is a hallmark of HPV-induced malignancy.

One of the most important and well-studied interactions of E6 is with the p53 tumor suppressor. The p53 protein plays a role in regulating key pathways in the cells, including cell-cycle regulation, activation of DNA repair pathways upon DNA damage, and induction of apoptosis. In the absence of HPV, p53 is regulated by the RING finger domain-containing ubiquitin ligase MDM2 (5). However, under stress, such as DNA damage, this pathway is inhibited, and p53 is stabilized and activated by a number of phosphorylation events, including phosphorylation at serine 15 after DNA damage. This phosphorylation induces a conformational change in p53 and disrupts its ability to bind to MDM2 (6). In HPV-infected cells, the interaction between p53 and MDM2 is disrupted, leading to the replacement of MDM2 by E6 in the process of ubiquitin-mediated degradation of p53 (7).

HPV E6 cannot induce p53 degradation on its own; it recruits the cellular E3 ubiquitin ligase UBE3A/E6AP through the LXXLL binding motif (8). This interaction induces a conformational change in E6, enabling it to effectively bind to p53 (9). The E6-E6AP complex then recruits and ubiquitinates p53, labeling it for proteasomal degradation. E6AP transfers ubiquitin from its C-terminal thioester cysteine bond to p53, which is then degraded. Recent studies have emphasized the importance of the E6AP interaction for E6 stability, as E6AP silencing in HPV-positive cells results in the rescue of p53 levels; however, E6 protein levels are greatly reduced, indicating that E6 stability is greatly dependent on the presence of E6AP (10).

E6 binding to E6AP induces a conformational change in E6AP structure, switching it between latent and active states (11). This conformational change not only allows E6 to interact with the HECT domain adjacent to the E2 binding site, but also positions E6 and p53 in close proximity to the catalytic residue of E6AP (12). The DNA-binding domain of p53 interacts with both the HECT domain of E6AP and the N-terminal zinc finger of E6, and undergoes ubiquitination by the complex (12). CryoEM studies on full-length E6AP, HPV 16 E6, and p53 revealed that HPV 16 E6 is sandwiched between the two proteins, providing support for the idea that E6 may serve as an adapter protein, transferring ubiquitin moieties to target protein p53 (13). This is further supported by a recent study that highlights E6AP as the “protector” of E6 ubiquitination. E6AP has a structured N-terminal ordered domain that has an alpha terminal helix, important for the interaction with the catalytically active HECT domain and E6 alpha helix. This interaction protects E6 from ubiquitination and displaces the charged E2 ubiquitin-conjugating enzyme UbcH7 (14).

Analysis of HPV-positive and -negative cervical and anal cancers showed that, while all HPV-positive cancers retained wild-type p53, some of the HPV-negative cancers had point mutations in the p53 gene (15). This indicates that loss of wild-type p53 is important for the progression of anogenital cancers and that, in the absence of E6-mediated degradation of p53, the cell employs other mechanisms to inactivate the protein. This is further supported by the evidence that the HPV-negative cervical cancer cell lines C33a and HT-3 have mutations in their p53 genes. C33a cells express p53 with an Arg to Cys mutation at codon 273, whereas HT-3 cells have a Gly to Val substitution at position 245 (16).

Virtually all studies in all models of cervical cancer have shown that not all p53 is degraded in HPV-positive cells. Detectable levels of the protein have been observed in cervical cancer cell lines, during viral infection, and in HPV-induced cervical lesions. This could be a result of E6*-mediated inhibition of p53 degradation: the alternatively spliced variant of E6 oncoprotein cannot induce p53 degradation and can bind to E6 and E6AP (17), blocking their ability to target p53. As E6* is abundantly present in HPV-positive cells, it suggests that there is a fine balance of p53 levels in transformed cells for optimal growth, and that there might be a functional reason for the maintained expression of the tumor suppressor protein. Further investigation is required to understand how HPV-mediated carcinogenesis might be modulated by this residual p53. In this study, we employed a genome-editing approach to knock out the residual p53 from HPV18-positive HeLa cells. We show that in the absence of p53, these cells exhibit profound defects in proliferation, metabolic activity, and invasion. These results demonstrate a gain-of-function (GOF) role for residual p53 in HPV-positive cancers and raise questions about the functional significance of the E6-p53 interaction.

## RESULTS

**Fig 1:**
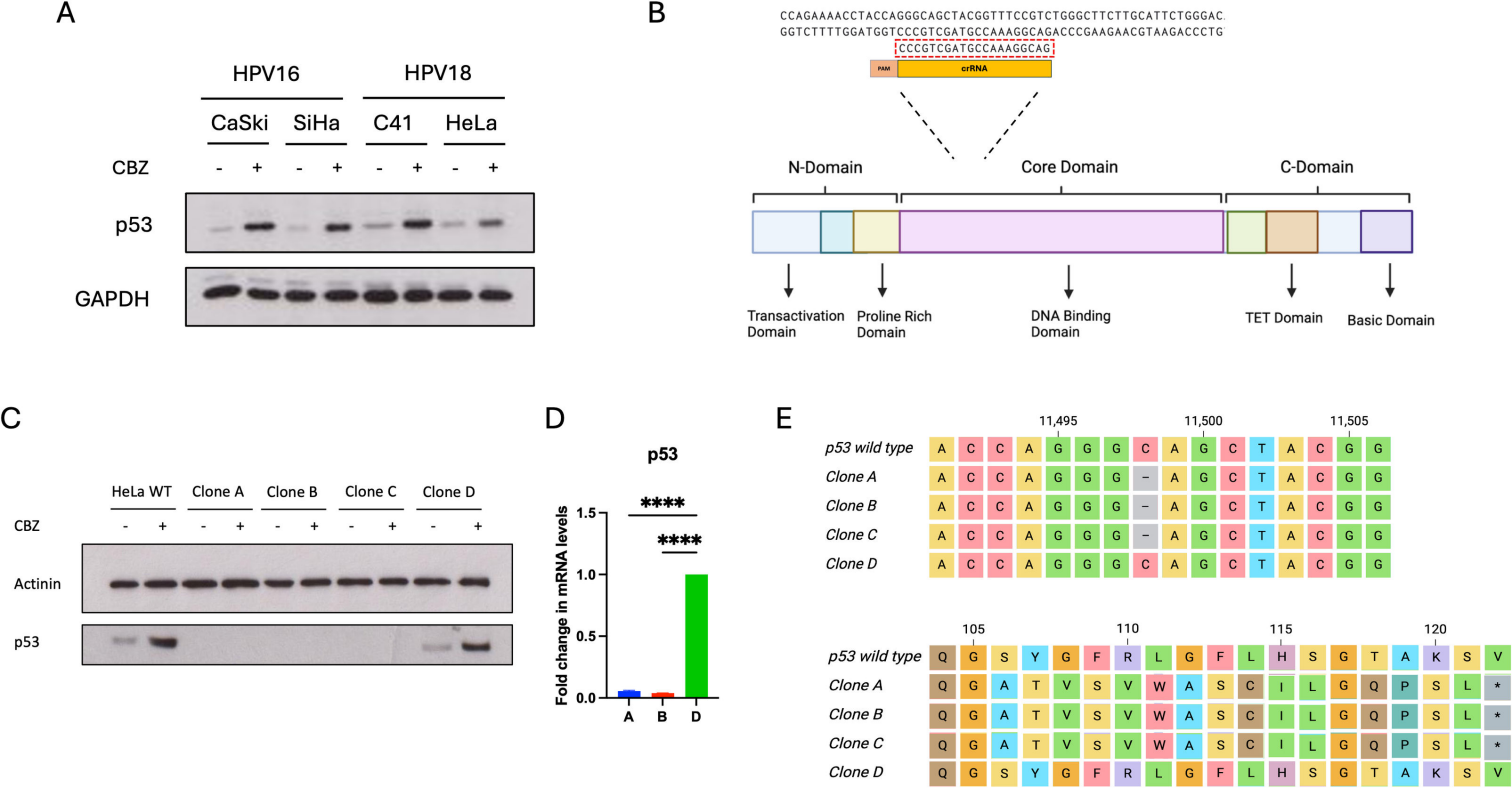
Generation of the p53 KO HeLa cell line. (**A**) Residual levels of p53 in HPV-positive cell lines: CaSki, SiHa, C41, and HeLa upon treatment with proteasomal inhibitor CBZ/MG132 (*n* = 3). (**B**) Design of CRISPR RNA (crRNA), targeting exon 4 of the p53 gene, using the IDT Alt-R HDR Design Tool (https://eu.idtdna.com/site/order/designtool/index/HDRDESIGN). The crRNA was conjugated to a transactivating CRISPR RNA (tracrRNA) with an ATTO 488 tag in a 1:1 ratio to form the guide RNA (gRNA). The gRNA was then incubated with the Cas9 protein to form the CRISPR Cas9 ribonucleic protein complex (RNP complex), which was electroporated into HeLa cells using the Amaxa Nucleofector Kit C and the Lonza nucleofector transfection 2b device. The cells were incubated for 1 hour and then sorted by FACS. The sorted cells were then subjected to single-cell cloning and further screening for the identification of p53-knockout cells. (**C**) Western blot analysis of screened p53 KO clones (*n* = 3). (**D**) mRNA levels of p53 in selected clones were analyzed to further verify p53 KO (*n* = 3). (**E**) Validation of genome editing with Sanger sequencing.

Despite the efficient degradation of p53 by HR HPV E6, residual p53 levels are observed in all HPV-positive cell lines (Fig. 1A), indicating that there might be a fine balance between p53 levels and HPV-mediated carcinogenesis. We hypothesized that the residual pool of p53 might have a functional role in the maintenance of the transformed phenotype, analogous to a “gain-of-function” mutation. To investigate this, we acquired fresh HeLa cells from ATCC and generated an HPV 18-positive p53 knockout (KO) HeLa cell line. This model would allow us to perform a series of phenotypic assays to determine whether this residual p53 has any growth-promoting activity in an HPV-transformed cell line. To select the clones with complete knockout of the p53 protein, we treated all single-cell clones with the protease inhibitor MG132 (CBZ) for 6 hours, and then harvested the cells and performed western blots to detect any rescued p53 protein. After testing 200 clones, we identified several that showed no p53 expression, even after proteasomal rescue, as shown in Fig. 1C. We additionally tested for truncated versions of the protein by using N-terminal specific p53 antibodies (data not shown) but did not observe any expression of the protein. We also selected a clone, clone D, that had undergone the selection process but still expressed p53 at levels similar to those seen in the parental HeLa cells. This was included as an additional control to ensure that any observed phenotypic differences were due to the knockout of the p53 protein and not a result of any off-target gRNA activity or cellular stress caused by Cas9 expression or FACS sorting and subsequent screening. All clones were then sequenced to check where and what mutation had occurred in the gene. PCR amplicons of exon 4 were analyzed and aligned to HeLa wild-type and clone D sequences. We observed a single nucleotide deletion in all three selected knockout clones that results in a stop codon due to the frameshift at residue 122 in exon 4, as shown in Fig. 1D. While the frequency of isolation of stable knockout clones was approximately 30%, the degree of editing varied amongst the clones due to the absence of the donor strand. The clones chosen were the most stable and genetically identical with respect to p53.

**Fig 2:**
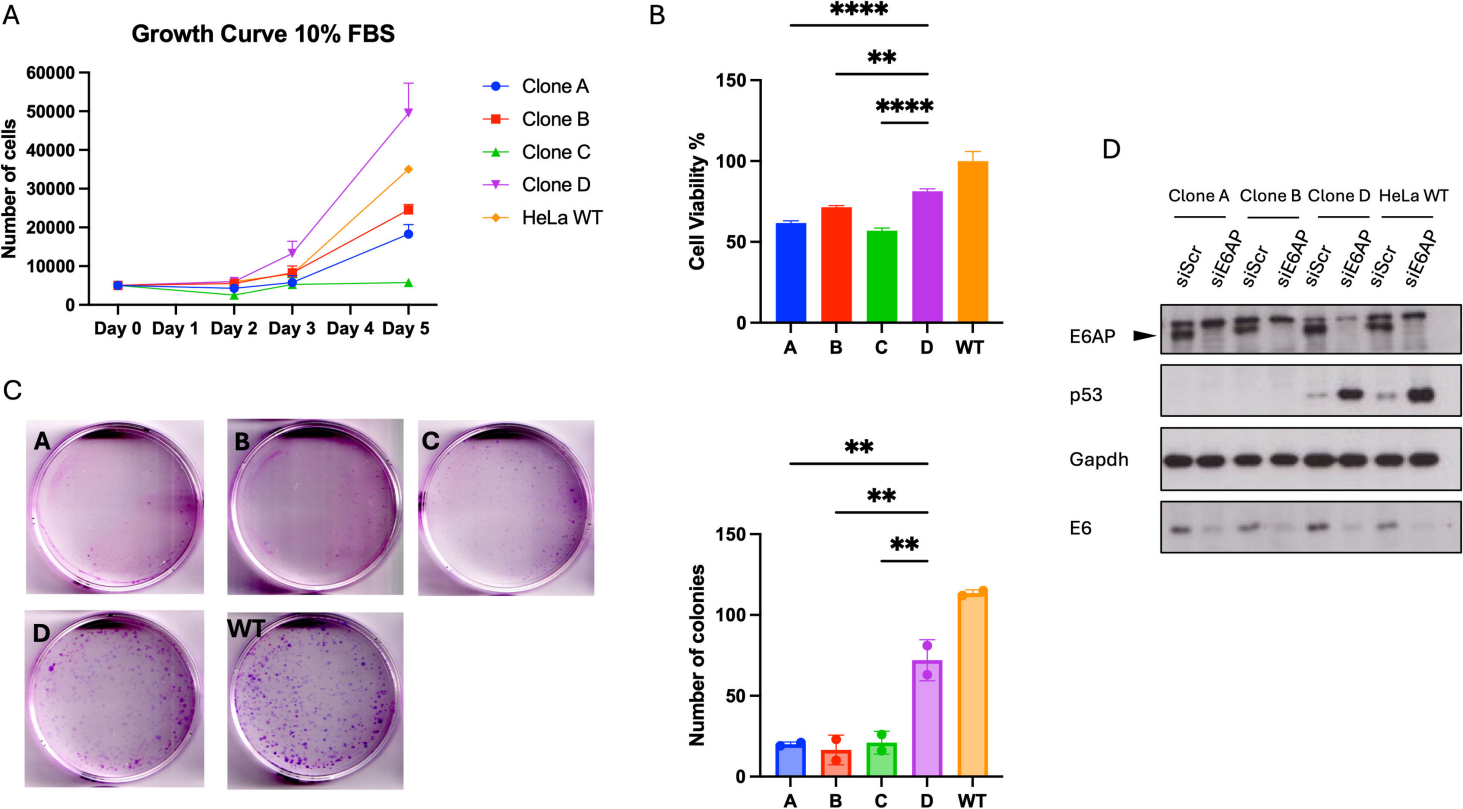
Loss of p53 correlates with low proliferation and survival. (**A**) p53 KO cells show a marked reduction in their ability to proliferate. The clones and controls were all seeded at 5,000 cells/well in a 12-well cell culture plate. The cells were harvested on days 2, 3, and 5 and counted using a hemocytometer (*n* = 4). (**B**) XTT assay shows decreased metabolic activity in the p53 KO cells. Five hundred cells/well for each cell line were seeded in 96-well plates. After 24 hours, XTT-labeling reagent was added to the plate for at least 5 hours. To determine the amount of formazan dye formed, the plate was analyzed using a scanning multiwell spectrophotometer (ELISA reader) at a wavelength of 490 nm. An increase in absorbance directly correlates with an increase in viable cells (*n* = 4). (**C**) Colony formation assay shows severely reduced cell survival in the absence of p53. All clones and controls were seeded in 60 mm cell culture dishes at 2,500 cells per plate and incubated for approximately 2 weeks. The colonies were then fixed and stained with 10% Giemsa and counted. Representative images from the colony formation assay and quantification of the number of colonies per sample are shown (*n* = 4). (**D**) E6 stability is not affected by p53 KO. p53 KO clones A and B, and p53 positive clone D, and HeLa WT were transfected with siScr (scrambled siRNA as a control) or siE6AP at a final concentration of 50 nM. After 72 hours, the cells were harvested, and western blots were performed. E6AP, p53, glyceraldehyde 3-phosphate dehydrogenase (GAPDH) (loading control), and E6 were detected using the respective endogenous antibodies (mAb DO-1 for p53 at concentrations shown in Table 1) (*n* = 3, *t*-test, **P value < 0.005, ****P value < 0.0001).

The first step was to determine whether these clones behaved any differently from p53 WT cells. Our initial observations indicated that the KO clones grew more slowly and took longer to reach confluence. Therefore, we performed a cell growth rate analysis using a proliferation assay. As shown in Fig. 2A, p53 KO clones grew much more slowly than the HeLa WT or clone D. This suggests that complete loss of p53 may be deleterious for HPV-positive cells and results in reduced rates of proliferation. To determine whether these slower-growing cells were viable and metabolically active, we performed an XTT cell viability assay. As shown in Fig. 2B, we observed that p53 KO results in fewer metabolically active cells than cells containing wild-type p53, suggesting that the residual p53 in HeLa cells might be significant for their high levels of metabolic activity. We next examined the cell survival and colony-forming abilities of these KO clones by performing a colony formation assay. Interestingly, we observed that in the absence of p53, the cells were unable to form robust colonies. These colonies were not only fewer in number, but also could not grow and expand in the plate as well as the wild-type p53 cells. Together, these assays indicate that the residual p53 might be functionally active in HPV-positive cells, potentially influencing pathways contributing toward the transformed phenotype.

As E6, E6AP, and p53 tend to exist in a complex in these transformed cells, we wanted to confirm that these growth differences were a result of the p53 knockout and were not influenced by E6 or E6AP levels or stability. Silencing E6AP has been shown to greatly reduce E6 levels, thereby rescuing p53 levels (10). Additionally, repression of E6AP in HPV-positive cells has been shown to induce cellular senescence, making the cells irreversibly growth-arrested (18). Thus, we performed siRNA-mediated silencing of E6AP in p53-positive and -negative clones and subsequently analyzed the E6 levels by western blotting. Our results show that E6AP and E6 levels remain unchanged in the p53 KO cells, compared with those in HeLa WT or clone D cells. However, loss of E6AP results in a dramatic decrease in E6 protein levels, regardless of the status of p53 (Fig. 2D). Restoration of p53 in clone D and HeLa WT cells further verifies the results of our silencing of E6AP, and the complete absence of p53 in the p53 KO cells further confirms the loss of p53 in these cells. These results suggest that the E6/E6AP dynamic is not affected by p53 levels, and the observed differences in proliferation and viability of the KO cells are most likely the direct result of p53 loss and are not due to any confounding effects upon the levels of E6 or E6AP expression.

**Fig 3:**
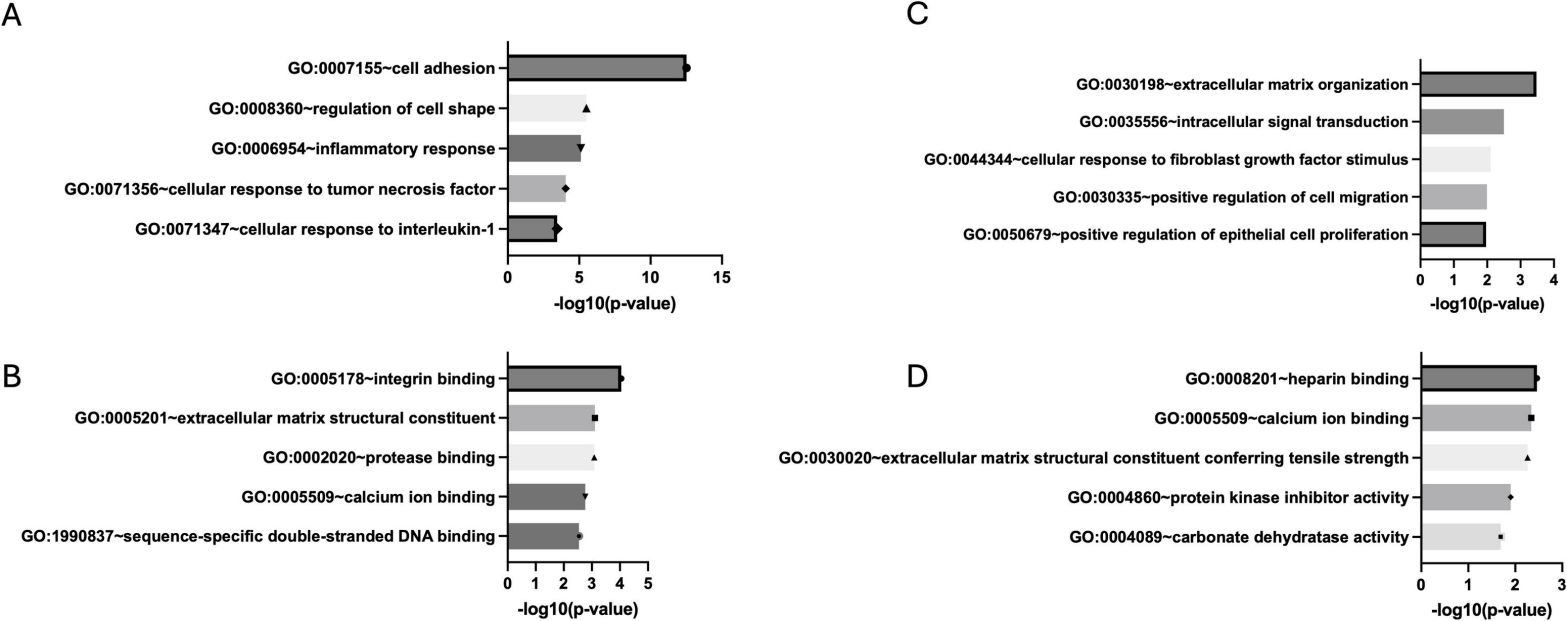
Transcriptome profiling reveals changes in gene expression programs between p53 KO and p53 WT HeLa cells. The top enriched (**A**) biological processes and (**B**) molecular functions of differentially expressed genes potentially related to p53 function, that were upregulated by log_2_(FoldChange) = 2 and had an adj *P* value < 0.05 between KO and WT cells. The top (**C**) biological processes and (**D**) molecular functions of equivalently downregulated differentially expressed genes.

We performed RNA sequencing analysis of the p53 KO clones to understand the transcriptomic profiles and pathways that may be affected by the loss of this residual p53. Clone A was used to represent p53 KO samples, while clone D was used as a WT control. We identified 1,310 genes with transcriptional changes with at least a log_2_(FoldChange) = 1 and a *P* value < 0.05. We began analyzing this data by performing functional annotation gene ontology (GO) analysis on our data set and restricting log_2_(FoldChange) = 2 to be more selective in our analysis. The differentially expressed genes with at least a log_2_(FoldChange) = 2 and a *P* value < 0.05 were analyzed using the DAVID bioinformatics software (https://david.ncifcrf.gov/). Figure 3A shows the top enriched terms associated with p53 function in the genes upregulated in p53 KO samples, compared with p53 WT. GO terms pertaining to cell adhesion and inflammation were upregulated in the absence of p53. The enriched molecular functions included integrin binding and extracellular matrix constituents (Fig. 3B). The top downregulated biological processes included those related to extracellular matrix organization, regulation of cell migration, and proliferation (Fig. 3C). The molecular functions included heparin binding, calcium ion binding, and the extracellular matrix structural constituent that confers tensile strength (Fig. 3D).

**Fig 4:**
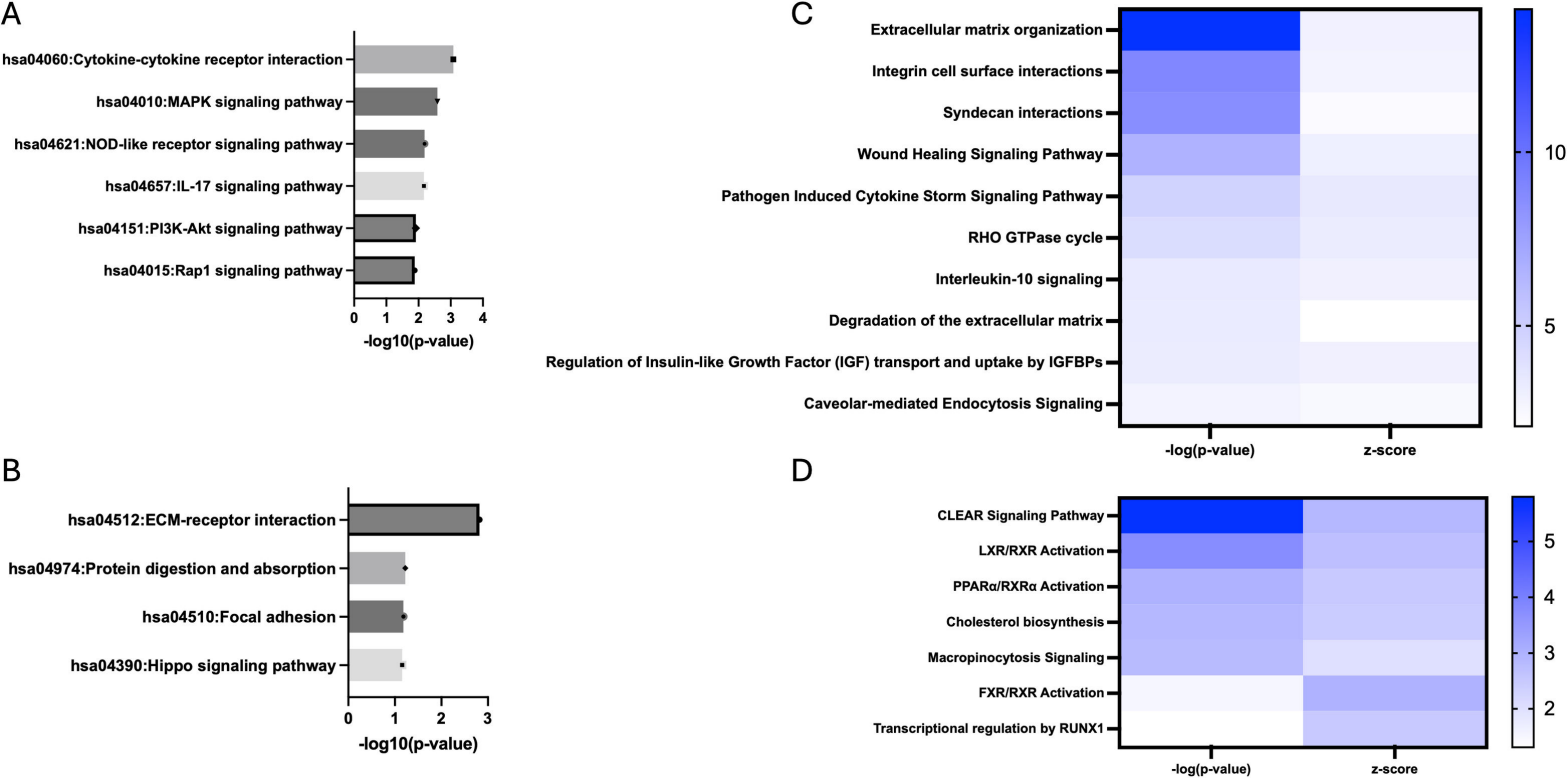
Functional pathway analysis of differentially expressed genes. KEGG pathway analysis of (**A**) upregulated genes and (**B**) downregulated genes. Ingenuity pathway analysis (IPA) of (**C**) upregulated genes and (**D**) downregulated genes shows consistent activation and inhibition of pathways.

Next, we contextualized this data further by performing a KEGG pathway analysis. As shown in Fig. 4A, enriched upregulated KEGG pathways included cytokine-cytokine receptor interaction and several signaling pathways. Our data indicate an activation of inflammation in the absence of p53, causing an increase in cell adhesion and stimulation of signaling pathways. KEGG analysis of the downregulated genes shows a downregulation of extracellular matrix (ECM)-receptor interaction, focal adhesion, and, interestingly, the Hippo signaling pathway. The observed differential expression of predominantly plasma membrane and extracellular matrix genes indicates that the loss of p53 causes a perturbation in cellular homeostasis.

These results were further validated by the canonical ingenuity pathway analysis (IPA). The software analyzed the levels of statistical significance and the degree of activation or inhibition of each pathway. The heatmaps in Fig. 4C and D show *P* values and z-scores for each pathway. A darker tile for the z-score indicates higher activation or inhibition. The most activated pathway was “pathogen-induced cytokine signaling pathway,” while the most inhibited pathway was “FXR/RXR activation.” FXR expression has been shown to inhibit cell proliferation and invasion in cervical cancer through a p53-mediated mechanism (19), further supporting the idea of a role for residual p53 in cervical cancer.

**Fig 5:**
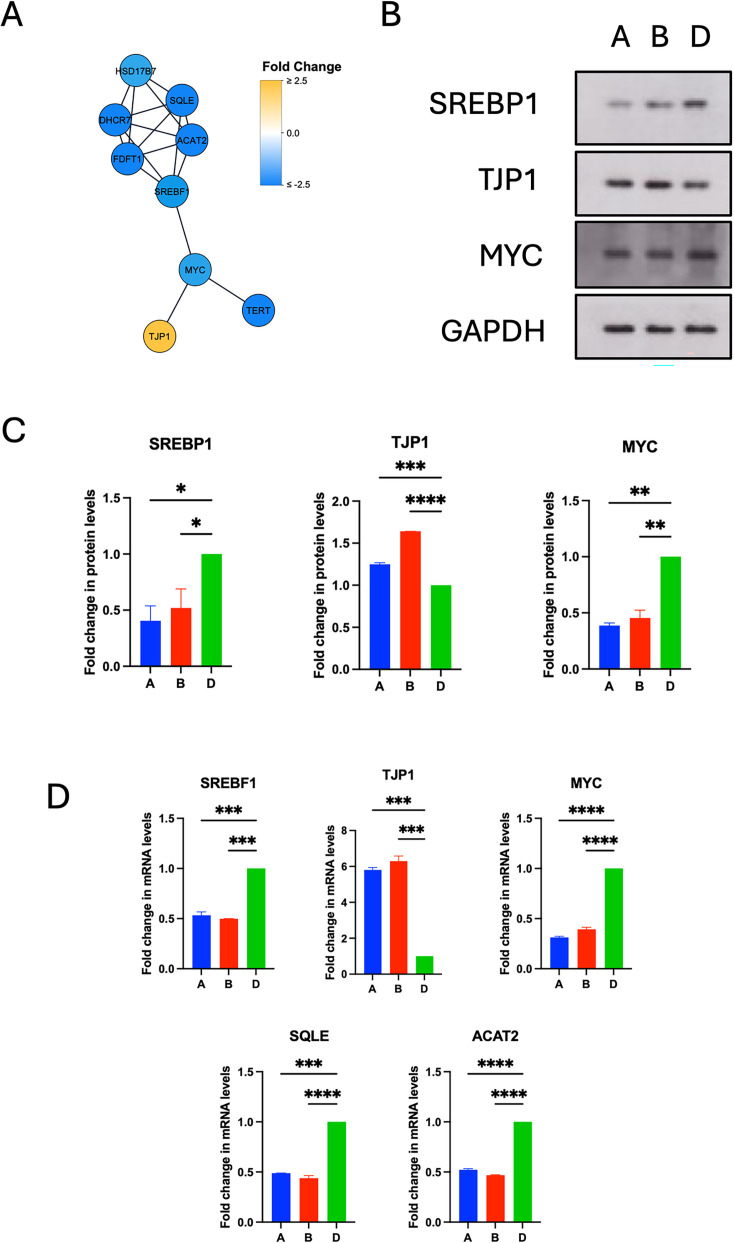
Differentially expressed mutant p53 target genes in the p53 KO HeLa cells. (**A**) Gene network analysis of mutant p53 target proteins that were differentially expressed in p53 KO cells. (**B**) Western blot analysis of a select few proteins from our analysis. (**C**) Quantification of the western blot analysis (*n* = 3). (**D**) qPCR validation of a few targets from the gene network using primer sequences shown in Table 2 (*n* = 3, *t*-test, **P <0.001, ***P =0.0001, ****P <0.0001).

Through our transcriptomic profile analysis, we identified several pathways and sets of genes that may contribute to the proliferation of these knockout cells. However, these did not explain the phenotypic changes we observed *in vitro*. Therefore, we analyzed our enriched pathways for any targets that might indicate a gain of function for residual p53, to explain the lack of proliferation of p53 KO HeLa cells. To this end, we compared our differentially expressed genes with published GOF mutant p53 targets. Sustained expression of GOF mutant p53 is known to increase the cellular levels of MYC (20, 21), and we observed that MYC levels were significantly downregulated in the absence of p53. Tert was also downregulated in our KO cells: mutant p53 can upregulate Tert expression in cells to give them a growth advantage (22). Mutant p53 has also been shown to upregulate the mevalonate pathway in breast cancer cells to modulate breast tissue architecture via the sterol regulatory element-binding protein (SREBP) transcription factors (23). We had already observed that cholesterol biosynthesis was inhibited in our IPA analysis. We therefore analyzed the enriched genes in this pathway and found that not only was SREBP1 (SREBF1 in our data set) significantly downregulated in the absence of p53, but other mevalonate pathway genes, such as SQLE, DHCR7, and ACAT2, also had reduced expression in the p53 KO cells. Mutant p53 is additionally involved in the disruption of cell polarity to promote epithelial-to-mesenchymal transition (EMT) and acquisition of a more aggressive cancer phenotype. In our analysis, we found an upregulation of TJP1 or ZO-1 in the p53 KO cells, which is targeted for degradation by GOF mutant p53 to promote EMT (24). We next confirmed the protein and mRNA levels of these targets to confirm their differential expression, as shown in Fig. 5B and C.

**Fig 6:**
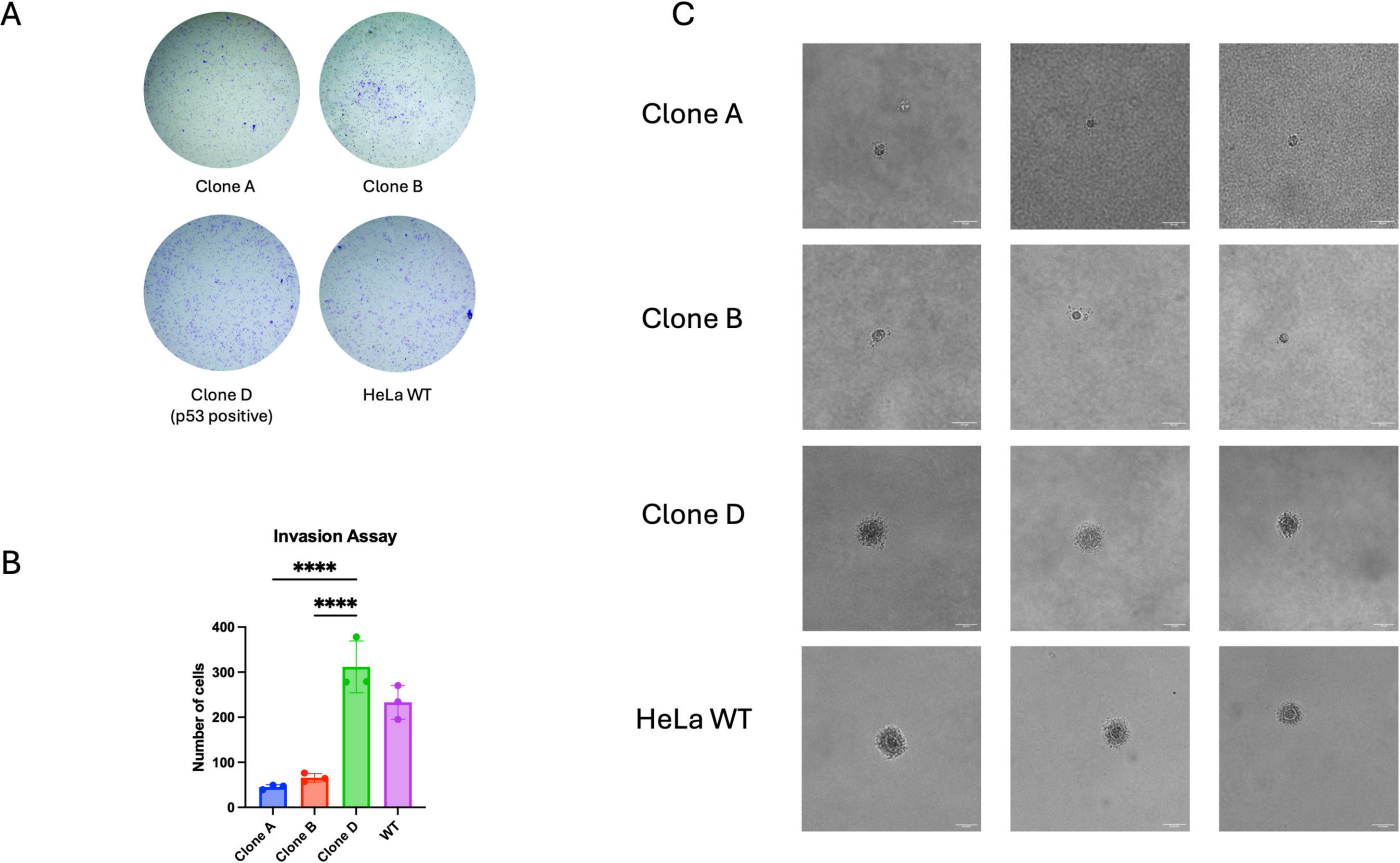
p53 KO clones lose their invasive capabilities. (**A**) Matrigel invasion assay shows loss of invasion in the p53 KO cells. The Corning invasion chambers were rehydrated using Dulbecco’s modified Eagle’s medium (DMEM) without any supplements. The cells were added to the upper chamber in serum-free medium, whereas the lower chamber contained medium with chemoattractant (10% FBS in this case). The chambers were incubated for 22 hours and then fixed with PFA, permeabilized with methanol, and stained with 0.005% crystal violet. The chambers were then washed, dried, and examined under a light microscope. (**B**) At least five random fields per chamber were obtained and counted, and the means were plotted in a bar graph (*n* = 3, *t*-test, ****P <0.0001). (**C**) Soft agar assay shows loss of invasion in the absence of p53. Cells were diluted in agar and normal DMEM to a final percentage of 0.4% agar. This mixture was plated on a solidified and cooled base layer of 1% agar. The wells were then fed with 1 mL of fresh DMEM every second day for a total of 21 days. The wells were then stained with 0.0005% crystal violet for 8 hours, washed with PBS, and examined using a light microscope and 20× objective lens (*n* = 3). Representative images from three independent experiments are shown.

As the transcriptomic analysis showed an enrichment of pathways related to cell surface proteins and the ECM, we tested the invasive capabilities of these cells. We performed a Matrigel invasion assay using Corning Matrigel invasion chambers. HeLa wild-type cells are highly invasive, as shown in Fig. 6A, and this phenotype is maintained in the control clone D. However, both p53 KO clones, A and B, had lost this characteristic and were unable to invade through the Matrigel. These findings suggest a potential role for residual p53 in the invasive capabilities of transformed cells.

As HeLa cells tend to thrive in soft agar and have been considered a positive control for tumorigenicity test by the WHO, we decided to perform a soft agar invasion assay. We observed that p53-positive cell lines were able to divide, grow, and invade into the matrix, and appeared in different planes throughout the gel. However, the p53 knockout clones were only visible in the same plane. This indicates that they are not able to invade through the soft agar and are also unable to divide and form robust colonies, as shown in Fig. 6C. The ability to proliferate in a non-adherent culture is a key feature of tumorigenic or malignant cells (25); thus, the loss of invasion in p53 KO cells suggests that residual p53 is important for the malignant properties of these HPV-transformed cells.

**Fig 7:**
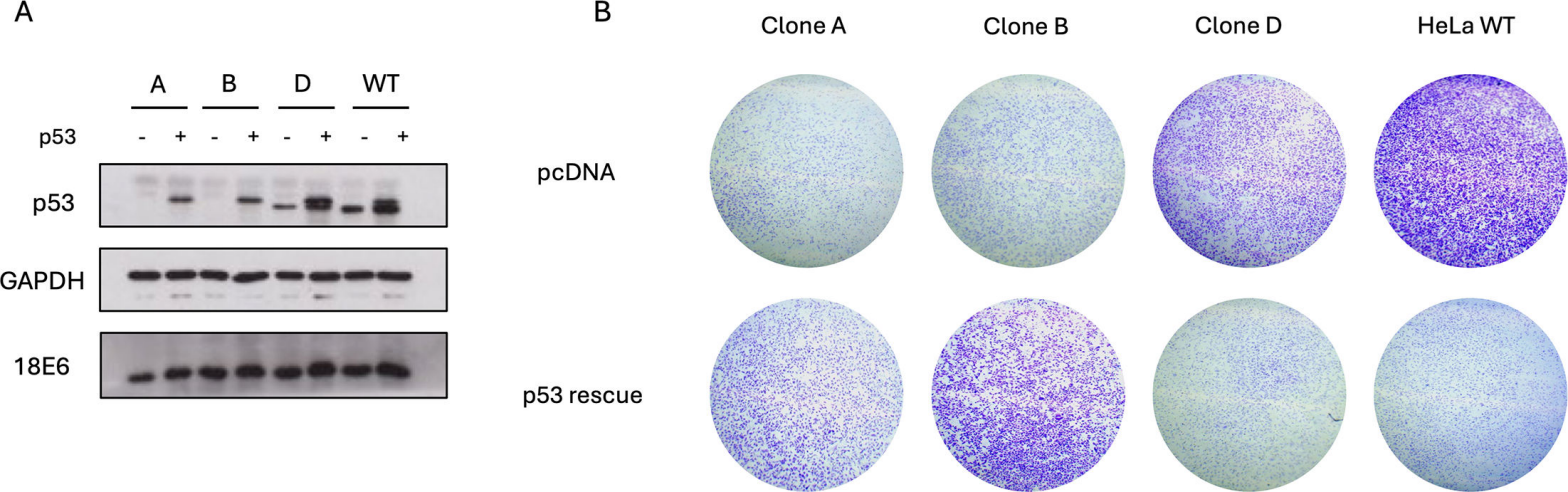
Exogenous p53 expression in p53-KO clones rescues the loss of invasive phenotype. Fifty nanograms of plasmid expressing Flag-tagged p53 was transfected into clones A, B, D, and HeLa WT cells, using Lipofectamine 3000 transfection reagent. (**A**) After 24 hours, the cells were harvested and analyzed by western blot to confirm p53 expression (*n* = 3). (**B**) They were then seeded in the upper chamber of Corning invasion wells in serum-free media. The wells were incubated in media with 10% FBS as a chemoattractant for 22 hours, after which the wells were washed, fixed, permeabilized, and stained with 0.0005% crystal violet overnight. The invasion chambers were dried and examined using a light microscope with a 4× objective lens.

To confirm that the loss of invasion observed above was a result of the p53 knockout, we decided to reintroduce p53 in these knockout clones (Fig. 7A). The results of this assay showed a clear increase in the invasive ability of the KO clones transfected with p53, compared with the control wells transfected with pcDNA. The observed invasion pattern of the p53-transfected KO cells was similar to that obtained for clone D transfected with pcDNA, or for HeLa WT, as shown in Fig. 7B. Interestingly, overexpression of p53 in the p53-positive cells, clone D, and HeLa WT, resulted in a decrease of invasion potential. These results suggest that a specific balanced level of p53 protein is required for optimal HPV-mediated carcinogenesis in transformed cells.

**Fig 8:**
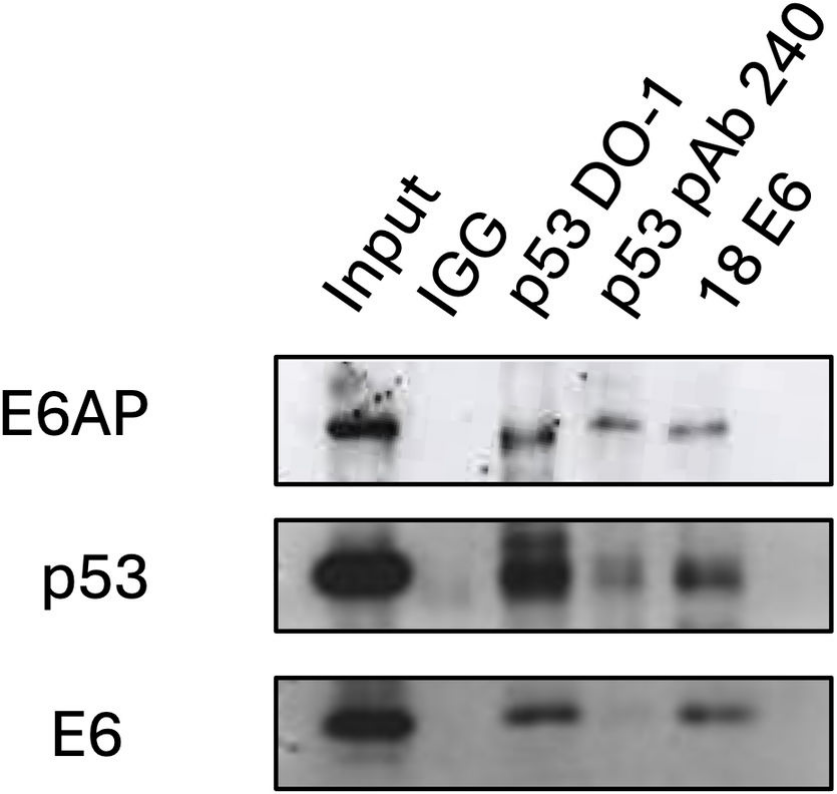
Antibody specific for conformationally-mutant p53 can immunoprecipitate E6AP from HeLa cells. Cell lysates of HeLa wild type were immunoprecipitated with anti-p53 DO-1, anti-p53 pAb240, and anti-HPV-18 E6 antibodies overnight and then incubated with protein A beads for 90 minutes at 4˚C. Immunoprecipitated complexes were then washed with lysis buffer and analyzed by western blotting for E6AP, p53, and 18E6 (*n* = 3).

It has been well established that HPV-positive cervical cancers do not contain point mutations in the p53 gene (16). However, conformational alterations could still be induced due to the presence of E6, which might contribute toward p53 inactivation and/or confer a “gain-of-function” phenotype. We performed a co-immunoprecipitation analysis using the anti-p53 antibodies DO-1, which detects wild-type p53, and pAb240, which detects conformationally mutant p53. Anti-HPV18E6 antibody was used as a positive control. Interestingly, we were able to immunoprecipitate E6AP with all three antibodies from wild-type HeLa cells. We attempted to confirm that this interaction is mediated by E6, and observed very faint binding with the p53 pAb240 antibody, potentially due to low levels of the viral protein in these transformed cells (Fig. 8). This assay suggests that E6 binding may induce a conformational change in p53 protein and change its functional abilities. The oncogenic functions observed here could be a result of this E6-induced conformational change.

## DISCUSSION

HPV E6-mediated degradation of specific host cell proteins is crucial for HPV-induced carcinogenesis; however, little is known about the oncoprotein’s continued contribution to the maintenance of the HPV-transformed phenotype. One of the most important functions of HR HPV E6 is the degradation of the tumor suppressor protein p53 (26) through the ubiquitin-proteasome pathway, via interaction with E6AP (27). In the absence of E6-induced degradation, p53 is inactivated through other mechanisms. Studies in HPV-negative cells have shown p53 inactivation via point mutations, further supporting the idea that loss of p53 function is vital for the development of anogenital cancers (15, 16). However, despite efficient degradation of the tumor suppressor protein (28), virtually all cervical cancer models show residual levels of p53 protein, suggesting that there might be a functional reason for the maintained expression of the tumor suppressor protein.

We performed genome editing to create a p53 KO, using HeLa cells as they tend to be quite robust and aggressive. We used a Cas9 ribonucleic protein (RNP) complex to ensure that expression of Cas9 was not a limiting factor and used an electroporator to nucleofect the cells, ensuring higher transfection efficiency. As p53 levels are already greatly reduced in these cells (4, 26), we treated the clones with the proteasome inhibitor MG132 to rescue any residual p53 present in the edited cells. Finally, we selected for further analysis those clones that had a verified inserted stop codon and no detectable levels of p53 protein.

Considering the role of p53 as a tumor suppressor and the induction of its degradation by HPV E6, we had hypothesized that these clones might grow faster in the absence of residual p53. Interestingly, however, the KO clones exhibited reduced proliferation, metabolic activity, and survival. This was interesting, as loss of p53 is generally associated with enhanced survival, and rescue of p53 levels in HPV-positive cells has been shown to induce cell cycle arrest (29, 30). We confirmed that these differences in behavior were indeed a result solely of the p53 deletion, and there was no effect on E6 protein levels or E6AP-mediated E6 stability through E6AP silencing.

There are many possible explanations for the observed differences in growth behavior, based on the many roles of the p53 protein, which plays a significant role in regulating the cell cycle and triggers cell cycle arrest upon DNA damage by inducing the expression of p21 (31). However, in the absence of p53 and the lack of this regulatory mechanism, the KO cells can undergo unregulated cell cycle progression. As p53 is also essential for genomic stability, the absence of p53 could lead to increased rates of mutation and chromosomal aberrations, resulting in genomic instability that might compromise growth and viability (32). Alternatively, this could also be explained by GOF p53. HPV-positive cervical cancer cells do not contain GOF mutant p53 in the classical sense (i.e., through one of the six hotspot mutations in the tp53 gene); we hypothesize that the formation of the E6-E6AP-p53 trimeric complex may induce conformational changes in the structure of the complexed p53 that mimic GOF mutant p53. It is known that mice expressing GOF p53 develop more aggressive and metastatic cancers (33, 34), and numerous studies have shown that conformational or contact mutations in p53 result in tumor cell growth-promoting functions.

GOF p53 can also induce the expression of some growth-related genes that are otherwise not targets of wild-type p53, such as MYC and cyclin B2 (22). Knockout of this p53 could result in a reduction or complete loss of cell growth and viability.

Our p53 KO and p53 WT cells were used as a model to detect differentially expressed genes (DEGs) and identify the potential associated signaling pathways. The top search terms included cell adhesion, integrin binding, and extracellular matrix structural constituent (ECM). Loss of cell adhesion is associated with tumorigenicity: during malignant transformation in the epithelium, cells lose their dependence on integrin-mediated attachment to the ECM and resulting signaling events (35). This results in a loss of adherens junctions that are important for cell-cell adhesion. These changes in the cell induce an EMT, which allows the tumor cells to adopt a more motile and invasive state (36). In the absence of p53, the observed upregulation in integrin binding would lead to enhanced ECM interactions. This would cause a loss in invasion and motility of these cancerous cells. Thus, p53 KO HeLa cells would tend to be less aggressive and metastatic in their phenotype. Loss of p53 is known to enhance the stemness of cells. This can also be observed in our model, where, in the absence of p53, stem cell characteristics are more pronounced, such as the loss of motility and increased cell-cell adhesion. Collectively, this is suggestive of residual p53 having pro-oncogenic functions in HPV-transformed cells and a role in maintaining cellular polarity and repressing cell adhesion to enhance the survival of transformed cells. Such observations highlight the importance of the ECM in the context of cancer aggressiveness and suggest that these pathways can be modulated to treat or cure HPV-driven cancers. Targeting the ECM structure or residual p53 may lead to tumor suppression or, ultimately, even a cure.

In the absence of p53, we observed a downregulation of ECM organization, intracellular signal transduction pathways, and ECM-receptor interaction. ECM-receptor interactions play an important role in the process of tumor shedding, adhesion, and movement. The role of ECM has been proven in several cancers: it has been shown to be increased in breast cancers (37), while the upregulation of ECM-receptor interactions can cause EMT in colorectal cancer cells (38), and can lead to metastasis in gastric cancer (39). HPV infection alters the ECM organization by dysregulating ECM molecules, such as matrix metalloproteinases, their inhibitors (TIMP1 and TIMP2), and claudins (reviewed in reference 40). Downregulation of these pathways and their genes indicates that residual p53 may be involved in their stability or transcription. The focal adhesion pathway is also downregulated in the absence of p53. In HPV-positive cervical cancer cells, downregulation of focal adhesion was shown to be vital for cellular transformation by HPV E6 (41). The p53-mediated further inactivation of this pathway is again suggestive of the role of residual p53 in regulating plasma membrane proteins and cell adhesion.

Inhibition of pathways associated with lipid metabolism and cholesterol synthesis was an interesting result seen in our p53 KO cells, as they are pathways modulated by GOF p53. In response to stress, wild-type p53 halts cell division and inhibits expression of SREBPs, which, in turn, downregulate the synthesis of lipogenic enzymes and cholesterol (42–44). GOF p53, on the other hand, induces expression of SREBPs and their target genes, such as SQLE and DHCR7, to maintain a supply of fatty acids and other lipids, which are vital for cell division. This is consistent with the increased demand for lipid proteins in rapidly proliferating cells (23). Downregulation of SREBP in our p53 KO cell line is further supported by the enrichment of the cholesterol biosynthesis pathway. Together, they suggest that in the absence of residual p53 in these transformed cells, lipids vital for cell division are not present, and these cells cannot support rapid proliferation.

Downregulation of these lipid metabolism pathways aligns with known GOF p53 functions. GOF p53 is associated with the upregulation of isoprenyl cysteine carboxyl methyltransferase (ICMT), a key regulator in the post-translational processing of intermediates generated by the mevalonate pathway (45). ICMT ablation is associated with hindered growth of Ras-driven breast cancers (46). This suggests that mutant p53 also modulates lipid metabolism for rapid cell growth by enhancing isoprenoid processing mechanisms. This further suggests that a pool of residual p53 may have oncogenic properties similar to those of GOF p53. This is further supported by the downregulation of other GOF p53 target genes, MYC and Tert. The role of MYC as a proto-oncogene, capable of promoting cell growth and tumorigenesis, is well established (47). GOF p53 upregulates MYC levels in cells and relies on it as a co-activation target (20). Treatment of head-and-neck squamous cell carcinoma (HNSCC) with PI3K inhibitors inactivates a MYC-dependent GOF p53 transcription network, making the cells more sensitive to conventional therapies (21). Increased telomerase activity is associated with cellular immortalization (48): in HPV-positive cells, TERT is activated by E6 through direct or indirect interaction with MYC or Sp1 (49), and E6 can also activate TERT expression by cooperating with NFX1-123 (50) or by downregulating the TERT repressors, NFX1-91 (51), p300 (52), and Maz (53). GOF p53 also upregulates TERT expression to give the cells a growth advantage (22). Downregulation of these GOF p53 targets in our p53 KO HeLa cells further supports the idea of a GOF p53 pool in these HPV-transformed cells.

As the RNA sequencing indicated a dysregulation of the plasma membrane and ECM, we investigated the invasive potential of these KO cells and found that they were much less invasive than the parental cells, suggesting that p53 may be modulating genes and proteins related to invasion. This is also consistent with our RNA sequencing analysis, where we observed upregulation of cell adhesion and integrin binding-related genes. We then performed soft agar assays to assess the anchorage-independent growth abilities and transformation potential of the KO cells and found that in the absence of p53, the cells could not invade through the soft agar and also lost the ability to form robust colonies; whereas HeLa cells expressing WT p53 proliferate well in soft agar and form clumps of cells (54).

We then confirmed that this loss of invasion was dependent on p53. We observed a rescue of invasive capabilities in the KO cells upon re-expressing p53. To our surprise, we observed a loss of invasion in our WT p53 control cells overexpressing p53. However, this observed loss of invasion could be explained by the accompanying enhanced integrin binding and ECM interactions. Enhanced ECM binding would cause a loss of motility of cells, making them less invasive. In HPV-positive HNSCC, restoration of p53 levels negatively impacts tumorigenicity and results in smaller, less invasive tumors *in vivo* (55). HeLa cells with enhanced p53 levels also display a loss of invasive phenotype. Depletion of this increased p53 using RNAi rescues the invasion phenotype of the transformed cells (56). Rescue and ectopic expression of WT p53 in breast cancers also reduces their ability to proliferate and invade, via downregulation of LDHA (57). p53 expression in colorectal cancer cells also significantly suppresses cell invasion and migration by inhibiting Fascin expression (58). This suggests that HPV-positive cells require a specific balance of p53 levels to maintain their transformed phenotype and retain their carcinogenic properties. This may be due to p53’s vital role as a transcription factor. While the damaged cells need to suppress p53 activity to promote cell division, the HPV oncoproteins may require interaction with p53 to enhance or ablate expression of target genes. Another major target of E6 with roles in cell migration and invasion is the PDZ domain-containing substrates (59). It is unlikely that interaction with them is affected by loss of p53 *per se*, as the sites of interaction are quite distinct. However, recent studies have shown that p53 can regulate E6 phosphorylation by DNA PK within the E6 PBM, and thereby modulate the E6-PDZ-14-3-3 interactions (60). It is conceivable that loss of p53 could also indirectly affect this signaling complex and thereby contribute to some of the phenotypes observed here.

In HPV-positive cervical cancer cells, p53 has no point mutations; however, conformational alterations in p53 could be induced upon its interaction with the E6/E6AP complex. Such changes in p53 structure could inactivate the protein and/or result in GOF activity. The formation of the E6/E6AP complex is a prerequisite for the recruitment and eventual ubiquitination of p53 (27, 61). However, upon the formation of the trimeric complex, E6 and E6AP undergo conformational changes that allow the optimal placement of all proteins in the ternary structure (9, 12). It is possible that this also induces a change in p53 structure that gives it novel properties. One such structural change is the exposure of residues 213–217 in the S7 beta sheet of the p53 protein (62, 63). In WT p53, this epitope is hidden by the S6 beta sheet and neighboring loops, but a single missense mutation in the DNA-binding domain of the p53 gene is enough to expose these residues (62). The resulting mutant conformation can then be detected by the p53 pAb240 antibody and can target genes that promote tumorigenesis. Our results showed that the conformation mutant-specific antibody and the total p53 antibody both co-precipitate E6AP in HeLa cells in an E6-dependent manner. These results suggest that residual p53 contains a pool of conformationally altered GOF p53. This is consistent with the findings of a study performed on cervical biopsies of patients with HPV-positive warts, CINs, and carcinomas. 86% of the samples were positive for pAb240 staining upon immunohistochemical analysis (64). A plausible explanation for this is proposed by Jo Milner in her conformation hypothesis. According to Milner, p53 can undergo conformational changes during cell growth. In its WT form, p53 can exist in two conformations: a suppressor form that helps control or stop growth and a promoter form that encourages cell growth and proliferation (65). WT p53 adopts its promoter form upon nuclear localization to promote cell division. The pAb240 antibody can detect this WT p53 promoter confirmation. However, it can also detect GOF mutant p53 as it shares the same conformation as the promoter form (65). This suggests that the E6/E6AP complex may induce a conformational change similar to that of the p53 promoter form and mutant p53. This could possibly explain the observed oncogenic functions of residual p53 in HPV-positive tumor cells.

In summary, this study demonstrates that residual p53 is functionally active in HPV-positive cells and may have GOF properties that allow the maintenance of the HPV-transformed phenotype. The transcriptomic profiling reveals differential expression of pathways pertaining to p53 tumor suppressive functions; it also provides evidence for GOF p53 roles that are further supported by the phenotypic assays.

## MATERIALS AND METHODS

### Cell culture

Cell lines HeLa, HeLa p53 knockout, and HaCat were grown in Dulbecco’s modified Eagle’s medium (DMEM) (GIBCO, #31885-023) supplemented with 10% fetal bovine serum (GIBCO, #1027-106), penicillin-streptomycin (100 U/mL), and glutamine (300 µg/mL) at 37°C in a humidified air incubator containing 10% CO_2_. The proteasome inhibitor was MG132 or CBZ at a 20 µM concentration (Z-L-Leu-D-Leu-L-Leu-al, Sigma Aldrich #C2211).

### Antibodies and immunoblotting

The list of antibodies used in this study is shown in Table 1.

**TABLE 1:** List of antibodies used

Antibody	Supplier	Species	Dilution
GAPDH	Santa Cruz Biotechnology	Mouse	1:4,000
HPV 18 E6	Santa Cruz Biotechnology	Mouse	1:500
p53 DO1	Santa Cruz Biotechnology	Mouse	1:1,000
p53 pAb240	Santa Cruz Biotechnology	Mouse	1:500
SREBP1	Santa Cruz Biotechnology	Mouse	1:500
TJP1	Santa Cruz Biotechnology	Goat	1:500
UBE3A	BD Biosciences	Mouse	1:500
Anti-actinin	Santa Cruz Biotechnology	Mouse	1:4,000
Anti-mouse HRP	Jackson ImmunoResearch 111-035-046		1:5,000
Anti-goat HRP	Dako		1:2,000
γ-Specific anti-mouse HRP	Jackson ImmunoResearch 115-035-071		1:5,000

### siRNA transfections

Cells were seeded at 40% confluence in a 6-well plate, and after 24 hours, they were transfected with appropriate siRNA. The siRNAs used in this study are as follows: E6AP (Dharmacon, SMARTPool #L-005137-00-0005) and Scramble (Dharmacon, #D-001700-01-20) at a final concentration of 50 nM, using Lipofectamine RNAMaxi (Life Sciences Technologies, #13778-150) according to the manufacturer’s instructions.

### Design of crRNA, gRNA transfection, and FACS sorting

A crRNA targeting exon 4 of the Tp53 gene was designed using the IDT Alt-RTM HDR Design Tool (https://eu.idtdna.com/site/order/designtool/index/HDRDESIGN) to create a p53-knockout cell line. A total of 100 µM crRNA (5′-GAC GGA AAC CGT AGC TGC CCG TTT TAG AGC TAT GCT-3′) was then conjugated to 100 µM Alt-R CRISPR-Cas9 tracrRNA ATTO 488 (IDT #816759) at 95°C for 5 minutes. The samples were allowed to cool down to 18°C–22°C for 5 minutes. The gRNA was then stored at −20°C for up to a year.

The gRNA was transfected as an RNP complex. A total of 3 µL of gRNA and 120 pmol of Cas9 protein were incubated for 15–20 minutes at 18°C–22°C. The HeLa cells were then transfected with the RNP complex using the Amaxa Cell Line Nucleofector Kit C, according to the manufacturer’s protocols. Nucleofected cells were incubated in suspension for an hour at 37°C in a humidified air incubator containing 10% CO_2_. The cells were washed once with PBS and resuspended in 5 mM EDTA (pH 8) in PBS for FACS (BD FACSAria II) sorting. Cells positive for ATTO 488 were sorted and cultured in limiting dilution in appropriate plates.

### Isolation, verification, and screening of CRISPR mutants

Sorted cells were immediately plated in a 96-well plate for single-cell cloning. Single-cell clones were selected, propagated, and harvested for the detection of p53 levels by western blotting. Cells without p53 were then subjected to genomic DNA isolation and PCR amplification of the targeted region using these primers (p53_F: GACGGAAACCGTAGCTGCCC and p53_R: CGGCCAGGCATTGAAGTCTCATGGA). The amplicons were then purified and sent for sequencing. The chromatogram trace files were subjected to analysis using the ICE software developed by Synthego (https://ice.synthego.com/#/).

### Determination of growth kinetics

Wild-type and mutant clones were seeded at 5 × 10^3^ cells in a 12-well cell culture plate in DMEM with 10% FBS or no FBS. Cells were harvested with trypsin, dispersed, and counted using a hemocytometer every second day until HeLa wild-type cells reached confluency. Cell counts were analyzed using GraphPad Prism 7.0 to generate a growth curve and to determine the saturation density of each clone.

### XTT cell viability assay

Clones were seeded at 1 × 10^3^ cells in a 96-well cell culture plate in DMEM with 10% FBS or no FBS. After 24 hours, 50 µL of the XTT labeling mixture (prepared by mixing 5 mL of the XTT labeling reagent with 100 µL of electron coupling reagent) (Roche, #11465015001) was added to each well and incubated for 5 hours at 37°C in a humidified air incubator containing 10% CO_2_. The formazan dye that formed was quantitated using a plate reader at the wavelengths of 490 and 630 nm. The measured absorbance directly correlates to the number of viable cells. The data were analyzed and graphed using GraphPad Prism 7.0.

### Colony formation assay

The cells were seeded at low confluence in 6 cm dishes in DMEM with 10% FBS or no FBS. Once the colonies were visible to the naked eye, they were fixed and stained with Giemsa. The colonies were counted using countPHICS software for ImageJ.

### Matrigel invasion chamber assay

Matrigel invasion chambers (Corning BioCoat Matrigel Invasion Chamber) were brought to room temperature and rehydrated in DMEM without FBS for 2 hours in a humidified CO_2_ incubator. Cells were seeded at 5 × 10^5^ cells in 200 µL of growth medium without FBS into the upper chamber. A volume of 750 µL DMEM with 10% serum was added to the lower chamber as a chemoattractant. After 22 hours, DMEM and any cells remaining in the upper chamber were removed by wiping with a cotton swab. Cells that had invaded the lower chamber were then fixed and stained for 20 minutes with 0.5% Crystal Violet in 5% glutaraldehyde. After washing away the excess stain with distilled water, the membrane was removed from the insert housing and placed on a microscope slide for imaging and analysis using a transmitted light microscope at 20× magnification. At least three fields per membrane were counted for each cell line. Invaded cell counts were analyzed using GraphPad Prism 7.0.

### RT-qPCR analysis

Cells were harvested, and RNA was extracted using the Tri reagent system (Sigma Aldrich #T9424) and subjected to reverse transcription using the Quantitect Reverse Transcription Kit (Qiagen #205311), according to the manufacturer’s protocol. qPCR was performed with Power SYBR Green PCR Master Mix (Applied Biosystems #4367659) using the CFX96 Real-Time PCR Detection System (Bio-Rad). The primers used are listed in Table 2.

**TABLE 2:** RT-qPCR primer sequences

Primer designation	Primer sequence
MYC fwd	5′-TACTGCGACGAGGAGGAGAA-3′
MYC rev	5′-CGAAGGGAGAAGGGTGTGAC-3′
Tert fwd	5′-GAGAACAAGCTGTTTGCGGG-3′
Tert rev	5′-AAGTTCACCACGCAGCCATA-3′
SQLE fwd	5′-AGTTCGCCCTCTTCTCGGA-3′
SQLE rev	5′-TTCCTTTTCTGCGCCTCCTG-3′
ACAT2 fwd	5′-AGGCTCAGATCCTGTGGTCAT-3′
ACAT2 rev	5′-TCAGACACATCTTCCGGAGC-3′
TJP1 fwd	5′-TCGGACAAAAGTCCGGGAAG-3′
TJP1 rev	5′-TCGGACAAAAGTCCGGGAAG-3′
SREBP1 fwd	5′-GCTCCCTAGGAAGGGCCGTA-3′
SREBP1 rev	5′-AAGTGCAATCCATGGCTCCG-3′
GAPDH fwd	5′-CCACCCATGGCAAATTCC-3′
GAPDH rev	5′-TCGCTCCTGGAAGATGGTG-3′

### Wound healing assay

Cells were treated with 10 µg/mL Mitomycin C. After 24 hours, they were scratched with a sterile Artline p2 pipette tip. The cells were then washed twice with PBS and photographed immediately, and after 24 hours. The decrease in area of the scratch was analyzed and quantified using the ImageJ and Prism programs.

### Soft agar assay

Three percent agarose was prepared in PBS and sterilized the day before setting up the assay. The 3% agar was then diluted in a 1:3 ratio with DMEM containing 10% FBS. A volume of 2 mL of the diluted agar was added to each well of a 6-well plate. After this had solidified, 0.4% agar containing 5,000 cells per condition was plated in triplicate. The agar plates were fed with 1 mL of DMEM with 10% FBS every other day until the end of the experiment.

### RNA extraction and sequencing

Cells were homogenized using the TriZol reagent (Sigma-Aldrich, T9424). RNA was extracted using the phase-separation technique after incubating the samples with chloroform. The RNA was precipitated with isopropanol overnight at −80°C. It was resuspended in RNase/DNase-free water. mRNA libraries were obtained by the Illumina TruSeq stranded mRNA library construction kit. mRNA libraries were sequenced using Illumina Novaseq 6000 for 100 bp paired-end sequencing (60 M reads).

Triplicate sequencing was performed for the two experimental conditions, followed by quality assessment of raw sequence files using FastQC (v 0.11.9) (66). Transcript quantification was then conducted using STAR (v.2.7.10b) (67), aligning reads to the Ensembl GRCh38 genome assembly. This process mapped sequencing reads to the reference genome and quantified gene expression levels, providing the foundation for subsequent analyses. The core analysis utilized the R package DESeq2 (68) to analyze gene count data, applying normalization and variance stabilizing transformation to facilitate expression comparisons. DEGs were identified based on adjusted *P* values < 0.05 and fold change thresholds [log_2_(FoldChange) = 2]. Functional annotation of these DEGs was performed using the clusterProfiler package (69) , querying databases such as Gene Ontology, KEGG, and Reactome. This annotation process provided insights into the biological context of observed gene expression changes. The final stages involved advanced interpretative techniques and visualization. Principal component analysis was applied to explore high-dimensional data properties. Network analysis and visualization were performed using the igraph package (70) and Cytoscape software (71) , accessed through the RCy3 package (72) . This comprehensive approach, combining statistical analysis with biological interpretation and visual representation, enabled a thorough examination of RNA sequencing data from initial quality control through to final functional interpretation of results.

### Statistical analysis

All experiments were performed at least thrice, and data are shown as mean and standard deviation of the mean. Statistical significance was calculated using the GraphPad Prism 7.0 software using the unpaired two-tailed Student’s *t*-test. A *P* value below 0.05 was considered statistically significant, and throughout, the *P* values have been defined as follows: *P < 0.05, **P < 0.005, ***P < 0.001, while “ns” represents a non-significant *P* value above 0.05.

For the quantification of protein levels from western blots, the films were scanned, and the intensity of bands was measured using ImageJ software. The final relative quantification values are the ratio of net band to net loading control (glyceraldehyde 3-phosphate dehydrogenase [GAPDH]).

## Data Availability

The RNA-seq data reported in this article hashave been deposited in NCBI’s Gene Expression Omnibus (GEO) and are accessible through GEO Series accession number GSE325258.
